# Effect of Packaging Method and Storage Environment on Activity of Magnesium Oxide and Mechanical Properties of Basic Magnesium Sulfate Cement

**DOI:** 10.3390/ma17153628

**Published:** 2024-07-23

**Authors:** Yuxiao Wu, Peini Shi, Qingsong Yang, Na Zhang

**Affiliations:** 1 School of Civil & Environmental Engineering and Geography Science, Ningbo University, No. 818, Fenghua Street, Ningbo 315211, China; 216002712@nbu.edu.cn (Y.W.); 18223443107@163.com (P.S.); 2311110085@nbu.edu.cn (Q.Y.); 2 Ningbo Key Laboratory of Energy Geostructure, Ningbo 315211, China

**Keywords:** active magnesium oxide, basic magnesium sulfate cement, storage environment, packaging method, mechanical strength

## Abstract

As one of the raw materials of basic magnesium sulfate cement (BMSC), the activity of light-burned magnesium oxide (MgO) has an important effect on the hydration rate, hydration products, and mechanical properties of BMSC. To reveal the influence of packaging method, storage environment, and storage time on the activity of MgO and the mechanical properties of BMSC, an experiment was conducted by using ordinary woven bags, peritoneal woven bags, and plastic and paper compound bags to store the finished BMSC and the raw materials (light-burned MgO, MgSO_4_·7H_2_O, fly ash, and a chemical additive) under the conditions of natural environment, sealed environment, and wet environment, respectively. Comparative analysis of the effects of packaging method, storage conditions, and storage time on the activity of MgO and the mechanical properties of BMSC was performed through the mechanical strength test of mortar specimens. The results showed that in a sealed environment, the loss of a-MgO content in light-burned MgO was minimized, which was more conducive to keeping the mechanical properties of BMSC stable. In the wet environment, the mechanical strength of BMSC was significantly reduced in the early stage (1 day) due to the significant reduction in the activity of MgO, and the mechanical strength of the finished BMSC and prepared BMSC after 120 days of storage was still lost, regardless of the packaging method. However, the storage environment and packaging method had relatively little effect on the late mechanical strength (28 days) of BMSC. It is advisable to use ordinary woven bags for packaging in natural and sealed environments as this is more economical for engineering applications. Plastic and paper compound bags are superior to ordinary woven bags and peritoneal woven bags in wet environments.

## 1. Introduction

Nowadays, many existing studies are focusing on the mechanical properties of BMSC, rather than paying much attention to how they maintain their mechanical properties under different storage conditions. This study investigated how the mechanical properties of BMSC are maintained in different storage environments using different packaging methods. This directly affects the usage of BMSC in real-world construction projects.

Magnesium oxysulfate cement (MOS) is an air-hardened magnesium cementitious material invented by Enricht in 1891 through the patent “Artificial stone or cement”, which is a MgO·MgSO_4_·H_2_O ternary cementitious system consisting of a-MgO powder and a certain concentration of MgSO_4_ aqueous solution, and the main hydration products are alkali salt hydrates xMg(OH)_2_·yMgSO_4_·zH_2_O [1,2,3]. MOS has lightweight, low-alkali, high-strength, fire protection, and corrosion-resistance properties, among others, and is superior to the performance of ordinary Portland cement. It is commonly used in the production of lightweight partition wall boards, decorative wall panels, fireproof core boards, and so on [4,5,6,7]. At the same time, MOS has the characteristic of not easily absorbing moisture and returning to halogen form, which is better than magnesium oxychloride cement, so it has aroused great interest from researchers. In addition, the raw material used for the production of BMSC, light-burned MgO, has low energy consumption, and magnesium sulfate heptahydrate is a by-product of industrial flue gas desulfurization or seawater salt production, which gives BMSC excellent social and ecological values such as energy-saving and emission-reducing abilities and green environmental protection, which is expected to become the core of the future cement and building materials industry as this is of great interest to the researcher and the user [8,9,10].

In 1980, Urwongse L. et al. found that the hydration products of MOS have 3Mg(OH)_2_·MgSO_4_·8H_2_O (3-1-8 phase), Mg(OH)_2_·MgSO_4_·5H_2_O (1-1-5 phase), and 5Mg(OH)_2_·MgSO_4_·3H_2_O (5-1-3 phase); the 1-1-5 and 5-1-3 phases are unstable phases at room temperature and the 3-1-8 phase is considered a stable phase at room temperature. Still, it is difficult to generate more than 50%, so MOS has low strength [11]. In 2005, Deng Dehua promoted the formation of new hydrate crystals by using an S-type additive, which greatly improved MOS performance. Still, the composition and crystal structure of the discovered hydrate were not determined in Deng’s research [12]. In 2012, Yu Hongfa’s group synthesized the pure phase of the new hydration product by using additive modification technology and cooperated with the German Max-Planck Laboratory, successfully analyzing this new crystalline phase as alkali salt hydrate 5Mg(OH)_2_·MgSO_4_·7H_2_O (5-1-7 phase), and named the modified MOS basic magnesium sulfate cement (BMSC) [13]. In 2013, Robert analyzed the 3-1-8 phase of the Mg(OH)_2_·MgSO_4_·H_2_O ternary system using powder diffraction technology and proved through thermodynamic analysis that the 3-1-8 phase is not a stable phase at room temperature [14]. Since then, BMSC with the 5-1-7 phase as the main hydration product has become a research hotspot in the magnesium building materials industry. At present, domestic and foreign studies on BMSC mainly focus on the effects of the composition ratio of raw materials, chemical additives, and mineral admixtures on its mechanical properties, water resistance, and microstructural properties [15,16,17,18,19,20,21,22,23,24,25,26]. Among them, light-burned MgO, as an important component of the raw material of BMSC cement, and its a-MgO content have an important influence on the performance of BMSC. Wu Chengyou et al. [27] investigated the effect of different a-MgO contents on the performance of BMSC and found that when the molar ratio of magnesium oxide to magnesium sulfate (n(MgO)/n(MgSO_4_)) is 5, higher MgO activity can produce a faster hydration reaction. Still, MgO activity that is too high is unfavorable to the formation of the 5-1-7 phase, which leads to a reduction in the compressive strength of the early BMSC. The late compressive strength of the BMSC was found to increase with the decrease in MgO activity because the 5-1-7 phase crystallized to a higher degree in BMSC prepared with low-activity MgO. Li Yanyan et al. [28] investigated the effect of MgO activity, water resistance, and carbonation properties on BMSC. BMSC was prepared with different activities of MgO, and it was found that the average pore size of the BMSC matrix increased with the decrease in MgO activity, which led to a loose structure of hardened cement paste. As a result, the mechanical strength of BMSC was low and the water resistance became poor.

Light-burned MgO powder with 80% MgO content was used to prepare BMSC, and the a-MgO content was measured to be about 55–68% by the hydration method [29,30]. a-MgO is an effective component capable of neutralization or hydration reactions in the MgO·MgSO_4_·H_2_O system. Usually, under certain molar ratio conditions, the higher the a-MgO content, the faster the hardening speed of BMSC slurry, and the better the mechanical properties. However, light-burned MgO powder is prone to deliquescence during storage. It leads to a decrease in a-MgO content, which affects the hydration reaction of BMSC and the formation of hydration product phases and affects the mechanical properties of BMSC. To better guide the production practice of BMSC and its products, this study investigated the effects of packaging method, storage environment, and storage time on the activity of MgO and the mechanical properties of BMSC [31]. Different bags were used to store the finished BMSC and the raw materials used for BMSC preparation under natural, sealed, and wet environment conditions. The effects of storage conditions, packaging methods, and storage time on the activity of light-burned MgO and the mechanical properties of the prepared and finished BMSC were analyzed by testing the a-MgO content and characterizing the mechanical strength of the finished BMSC and prepared BMSC. The results of this study can provide important data support for the production practice of BMSC and its products and have important theoretical and application value.

## 2. Materials and Method

### 2.1. Raw Materials

The experimental light-burned MgO powder was obtained from Liaoning Huafeng Magnesium Industry Co. Ltd., and the a-MgO content was measured to be 64.4% (MgO content 80%) by the hydration method (Liaoning Suizhong Power Generation Co. Ltd., Huludao, China). Class I fly ash (fly ash, FA) and magnesium sulfate heptahydrate (MgSO_4_·7H_2_O) were produced by Shandong Yongxin Chemical Industry Co. Ltd., Heze, China (industrial-grade), and the chemical composition is shown in Table 1. The chemical additive citrate (analytically pure) was obtained from Aladdin (Shanghai) Reagent Co., Ltd., Shanghai, China.

### 2.2. Experimental Methods and Test

**Figure 1:**
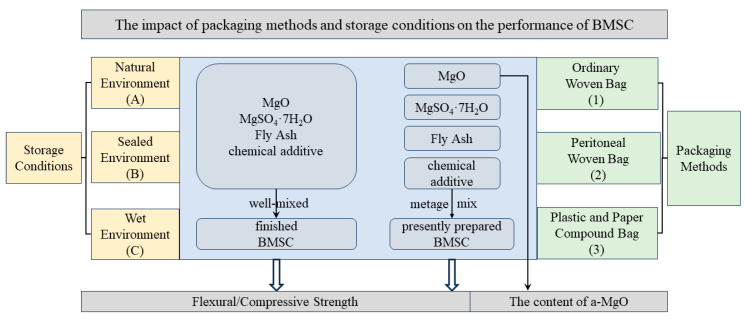
Schematic representation of the test method.

The granular MgSO_4_·7H_2_O was pulverized to about 200 mesh (sieve residue 9.6%) using a pulverizer. The molar ratio of a-MgO: MgSO_4_·7H_2_O was 8:1, the dosage of fly ash was 50% of the mass of MgO, and the dosage of the chemical additive was 0.2% of the mass of MgO. All the raw materials were uniformly mixed by a mixer to prepare 52.5R-grade BMSC-, i.e., 52.5R-grade finished BMSC cement. The finished BMSC was packaged in ordinary woven bags, peritoneal woven bags, and plastic and paper compound bags. The three kinds of packaged BMSC were stored in a natural environment (A), sealed environment (B), and wet environment (C) for 120 days, and samples were taken at 30-day intervals to test the mechanical strength of the mortar specimens. At the same time, each raw material, including light-burned MgO, MgSO_4_·7H_2_O, FA, and the chemical additive, was stored in different environments using the three packaging methods and taken out at the same time to prepare BMSC in the same proportions, i.e., prepared 52.5R-grade BMSC. Storage environment A: In the laboratory (Shenyang) from February to June, with the temperature between 18 °C and 25 °C and the humidity from 30% to 60%; B: the same laboratory, in a sealed bucket with desiccant; C: the same laboratory, with the use of a zinc sulfate saturated solution (with the theoretical standard relative humidity of 90%) to control the relative humidity of the film frame sealing space, with measured humidity in the confined space ranging from 75% to 85%. The experimental method is shown schematically in Figure 1 below, and the samples were labeled as shown in Table 2.

The experiments were conducted to test the activity of a-MgO content in light-burned MgO using the hydration method. According to Chinese national standards for the Common Portland Cement [32] and Test Method of Cement Mortar Strength (ISO Method) [33] cement mortar specimen preparation method and test method, a BMSC mortar specimen (shaking table molding) was prepared under the conditions of a mortar paste of 1:3 and a water–cement ratio of 0.36 (mortar fluidity of 180 ± 2 mm). After mixing, the slurry was poured into a 40 × 40 × 160 mm steel mold and then de-molded after 24 h of maintenance at a temperature of 20 ± 2 °C and a relative humidity of 60 ± 5%. This was then maintained until it had aged. The flexural and compressive strength of the BMSC mortar specimens was tested by the TYE-300D cement mortar flexural and compressive testing machine, and the loading rates were 50 N/s and 2400 N/s, respectively.

## 3. Results and Analysis

### 3.1. Influence on the Activity of MgO

**Figure 2:**
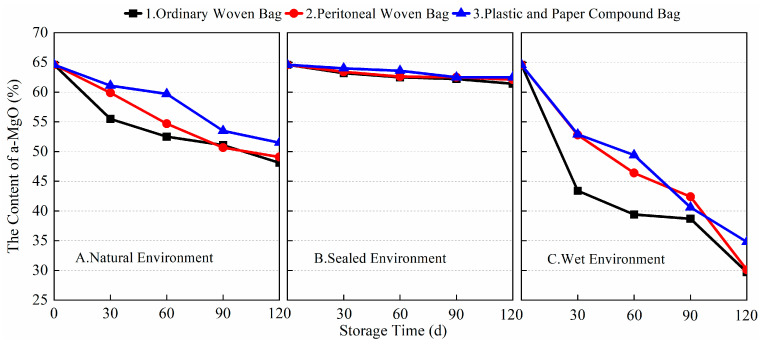
Variation of a-MgO content with storage time under different packaging and storage conditions.

The a-MgO content, as an important indicator characterizing the ability of light-burned MgO to react with magnesium salt solutions such as magnesium sulfate and form hydration products, had a significant impact on the performance of the BMSC. During storage, a-MgO is prone to moisture absorption and conversion to Mg(OH)_2_ or carbonization and conversion to MgCO_3_, which reduces the a-MgO content in light-burned MgO. Therefore, the packaging method, storage environment, and storage time become important factors affecting the activity of MgO and the performance of BMSC. As shown in Figure 2, the changes in a-MgO content in light-burned MgO with different packaging methods, storage environments, and storage times were measured by the hydration method. As can be seen from the data, the a-MgO content gradually decreased with the extension of storage time. At 120 days of storage, the a-MgO contents of A1, A2, A3, B1, B2, B3, C1, C2, and C3 were 48.1, 49.1, 51.5, 61.4, 62.1, 62.5, 29.7, 30.1, and 34.8%, respectively. The dry and sealed environment could isolate the wet air, which was favorable for the storage of light-burned MgO, and the activity of MgO was only reduced by about 2.0% for 120 days. In a wet environment, no matter which packaging method was used, the MgO activity reduction was more obvious, decreasing by about 30.0% after 120 days. Among the three packaging methods, plastic and paper compound bags prevented the reduction in MgO activity better than peritoneal woven bags and ordinary woven bags. Therefore, a dry environment should be ensured as much as possible during the production, transportation, and use of light-burned MgO powder, which is conducive to maintaining MgO activity and thus controlling the production of BMSC and the quality of its products more effectively.

### 3.2. Effect of MgO Activity on BMSC Setting Time

**Figure 3:**
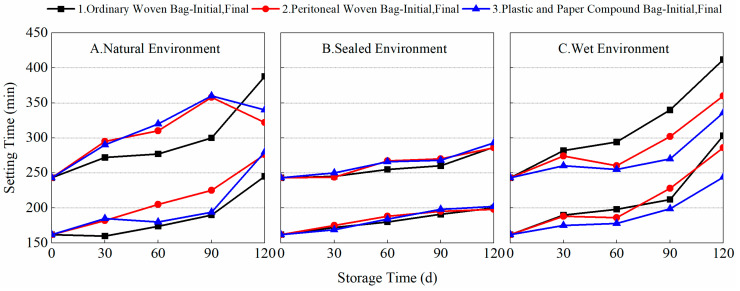
Variation of setting time of finished BMSC with storage time under different packaging and storage conditions.

**Figure 4:**
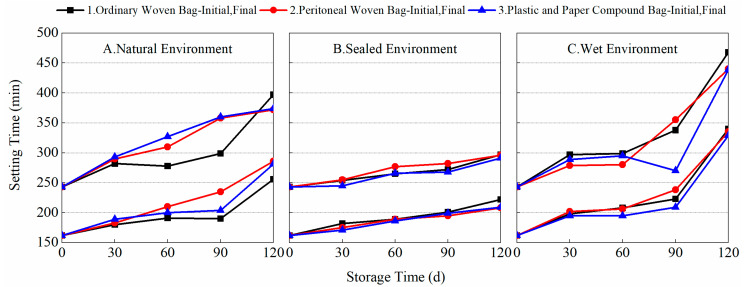
Variation of setting time of the prepared BMSC with storage time under different packaging and storage conditions.

Setting time, as an important indicator of the hydration rate of cement, is an important guide for evaluating the construction efficiency of cement-based materials. However, in BMSC, the fineness of raw-material light-burned MgO and its a-MgO content have a significant effect on the rate of setting and hardening of BMSC. Experiments were conducted to test the setting time of the finished BMSC and prepared BMSC stored using different packaging methods in different environmental conditions, as shown in Figure 3 and Figure 4.

Figure 3 shows the variation in the setting time of the finished BMSC cement with storage time for different packaging methods and storage environments. From the data, it can be seen that with the extension of storage time, the finished BMSC cement’s initial and final setting times gradually increased. When stored for 90 days, the initial setting times of A1, A2, A3, B1, B2, B3, C1, C2, and C3 were 190, 225, 194, 191, 195, 198, 212, 228, and 199 min, respectively, and the final setting times were 300, 358, 360, 260, 270, 268, 340, 302, and 270 min, respectively. Combined with the data in Figure 2, it can be found that the length of the setting time of the finished BMSC was closely related to the content of a-MgO. Under the sealed environment, a-MgO was not easily converted to Mg(OH)_2_ by moisture absorption or to MgCO_3_ by carbonization, and the content of a-MgO was relatively high, so the changes in the setting times of the BMSC were small, and there was not much difference between the three packaging methods. In the natural environment, due to the relatively significant decrease in the content of a-MgO, the BMSC setting times were significantly prolonged. This would have a greater impact on construction efficiency. In the wet environment, the use of plastic and paper compound bags was more conducive to preventing reductions in MgO activity and thus reducing the extension of the setting time of BMSC cement. As shown in Figure 4, in different storage environments, the variation rule of the setting time of the prepared BMSC cement with storage time using different packaging methods was similar to that of the finished BMSC. These results show that the setting time of BMSC is closely related to the content of a-MgO in light-burned MgO. The adoption of a suitable packaging method can effectively prevent the decrease in a-MgO content in light-burned MgO and reduce its effect on the hydration reaction of BMSC. It is more favorable to store MgO in an ordinary woven bag in a natural environment, and it is more favorable to use plastic and paper compound bags in a wet environment.

### 3.3. Effects on the Mechanical Properties of the Prepared BMSC

**Figure 5:**
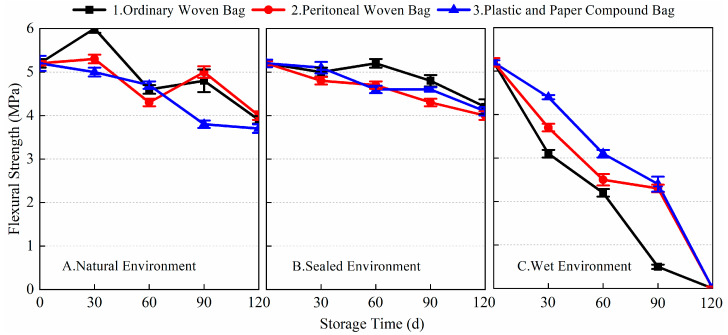
The 1-day flexural strength of the prepared BMSC with different packaging methods and storage conditions.

**Figure 6:**
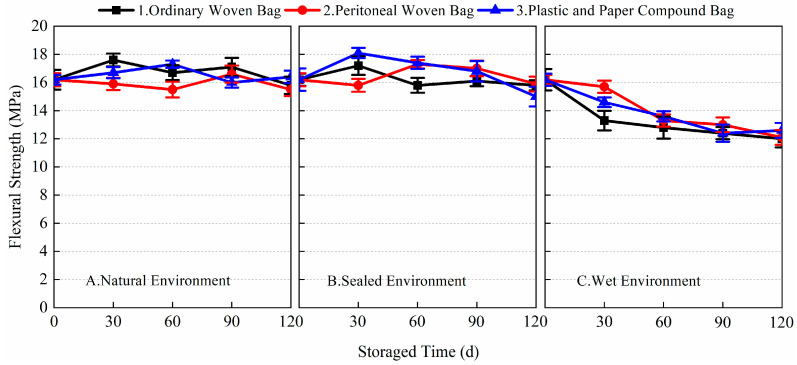
The 28-day flexural strength of the prepared BMSC with different packaging methods and storage conditions.

**Figure 7:**
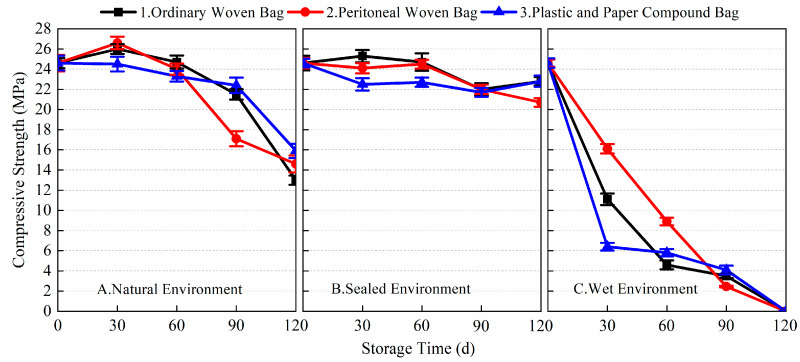
The 1-day compressive strength of the prepared BMSC with different packaging methods and storage conditions.

**Figure 8:**
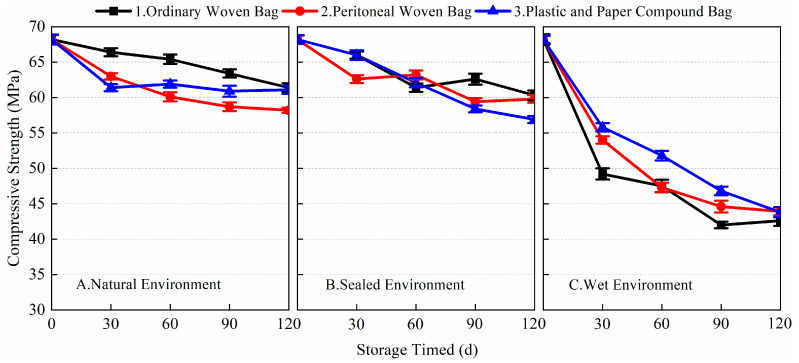
The 28-day compressive strength of the prepared BMSC with different packaging methods and storage conditions.

Figure 5, Figure 6, Figure 7 and Figure 8 show the data for the 1-day and 28-day mechanical strength of the prepared BMSC mortar specimens using different packaging methods and storage environments with the change in storage time. The 1-day flexural and compressive strengths of the blank sample (stored for 0 days) were 5.2 and 24.6 MPa, respectively; the 28-day flexural and compressive strengths were 16.2 and 68.2 MPa, respectively.

From the 1-day and 28-day flexural strength data of the prepared BMSC mortar specimens in Figure 5 and Figure 6, it can be seen that different packaging methods and storage conditions had a greater impact on the 1-day flexural strength of the prepared BMSC, while the impact on its 28-day flexural strength was relatively small. After 120 days of storage, the 1-day flexural strength of the presently prepared BMSC stored using the three packaging methods under the three storage conditions decreased significantly. The largest decrease in 1-day flexural strength was observed for the prepared BMSC in a wet environment. The flexural strengths corresponding to the three packaging methods all decreased by 100% compared with the blank samples; in the natural environment, the decrease in the 1-day flexural strength of the prepared BMSC was relatively small. The corresponding flexural strengths of the three packaging methods decreased by 25, 23.1, and 28.8%, respectively, compared with the blank samples. It is noteworthy that there was even a slight increase in the flexural strength after 30 days of storage; the smallest decrease in 1-day flexural strength was observed for the prepared BMSC in a sealed environment. The corresponding flexural strengths of the three packaging methods decreased by 19.2, 23.1, and 21.2% compared to the blank samples, respectively. The 1-day flexural strengths of the prepared BMSC-A1, A2, A3, B1, B2, B3, C1, C2, and C3 were 3.9, 4.0, 3.7, 4.2, 4.0, 4.1, 0, 0, and 0 MPa, respectively, after storage for 120 days. These results suggest that in the natural and sealed environmental conditions, the early flexural strength of the prepared BMSCs stored in ordinary woven bags was better than those stored in the peritoneal woven bags and plastic and paper compound bags, whereas the early flexural strength of the prepared BMSCs stored in plastic and paper compound bags was better than those stored in the peritoneal woven bags and ordinary woven bags in wet environmental conditions.

Figure 6 shows the data of the 28-day flexural strength of the prepared BMSC with storage age for three packaging methods under different storage conditions. As shown in the figure, there was almost no decrease in the 28-day flexural strength of the prepared BMSC stored for 120 days in natural and sealed environments compared to the blank samples. The flexural strengths corresponding to the three packaging methods in the natural environment decreased by 2.5, 4.3, and −1.2% compared to the blank samples, respectively. The flexural strengths of the three packaging methods in the sealed environment decreased by 2.5, 1.9, and 7.4%, respectively, compared with the blank samples. In the wet environment, the 28-day flexural strength of the prepared BMSC decreased gradually with the extension of storage time, but the decrease was smaller, and the corresponding flexural strengths of the three packaging methods decreased by 25.9, 25.3, and 22.2%, respectively, compared with the blank samples. The experimental results showed that the storage conditions and packaging methods had a significant effect on the early flexural strength of the prepared BMSC; the effect on the 28-day flexural strength of the prepared BMSC under natural and sealed environment conditions was relatively small, but the effect on the 28-day flexural strength of the prepared BMSC in a wet environment was more obvious.

Figure 7 and Figure 8 show the 1-day and 28-day compressive strength data of the prepared BMSC mortar specimens under different storage and packaging conditions. From the data, it can be seen that different storage environments and packaging methods had obvious effects on the 1-day and 28-day compressive strength of the prepared BMSC. As shown in Figure 7, it can be found that the loss of 1-day compressive strength of the prepared BMSC stored in a sealed environment was the smallest compared with that in a natural environment and a wet environment, and the 1-day compressive strength of the prepared BMSC gradually decreased with the extension of storage time. After 120 days of storage, the compressive strengths corresponding to the three packaging methods decreased by 7.3, 15.9, and 7.3%, respectively, compared with the blank samples. Under the conditions of natural and sealed environments, the early compressive strength of the prepared BMSC decreased more slowly in 0~90 days. The 1-day compressive strength of the prepared BMSC in a natural environment decreased more significantly in 90~120 days; in the sealed environment, the 1-day compressive strength of the prepared BMSC decreased still more slowly, and the performance of the BMSC packaged in ordinary woven bags and plastic and paper compound bags was superior. After 120 days of storage, the decreases in the BMSC 1-day compressive strength using the three packaging methods in a natural environment were 47.2, 40.7, and 35.4% respectively; under wet environmental conditions, the early compressive strength of the prepared BMSC decreased significantly after 30 days of storage, and the losses of BMSC 1-day compressive strength of the three packaging methods were 54.9, 34.6, and 74.0%, respectively. With the prolongation of storage time, the compressive strength decreased significantly, and the mechanical strength was no longer available after 120 days of storage.

Figure 8 shows the change in the 28-day compressive strength of the prepared BMSC with storage time under different storage and packaging conditions. In a natural environment and sealed environment, the 28-day compressive strength of the prepared BMSC mortar specimens decreased more gently, while the decrease in the 28-day compressive strength of the prepared BMSC in a wet environment was more significant. In a natural environment, the 28-day compressive strength losses of the prepared BMSC mortar specimens A1, A2, and A3 stored for 90 days under three different packaging conditions were 7.0, 13.9, and 10.7%, respectively, compared to the blank samples; in a sealed environment, the 28-day compressive strength losses of the prepared BMSC mortar specimens B1, B2, and B3 stored for 90 days under three different packaging conditions were 8.2, 12.9, and 14.4%, respectively, compared to the blank samples; and in a wet environment, the 28-day compressive strength losses of the prepared BMSC mortar specimens C1, C2, and C3 stored for 90 days under three different packaging conditions were 38.4, 34.6, and 31.4%, respectively, compared to the blank samples. The results showed that the mechanical strength of the prepared BMSC stored in ordinary woven bags was better than that of the prepared BMSC stored in peritoneal woven bags and plastic and paper compound bags in natural and sealed environments. In a wet environment, the mechanical strength of BMSC obviously decreased no matter which packaging method was used, and the effect of the peritoneal woven bag was better than that of the other two packaging methods.

### 3.4. Effects on the Mechanical Properties of the Finished BMSC

**Figure 9:**
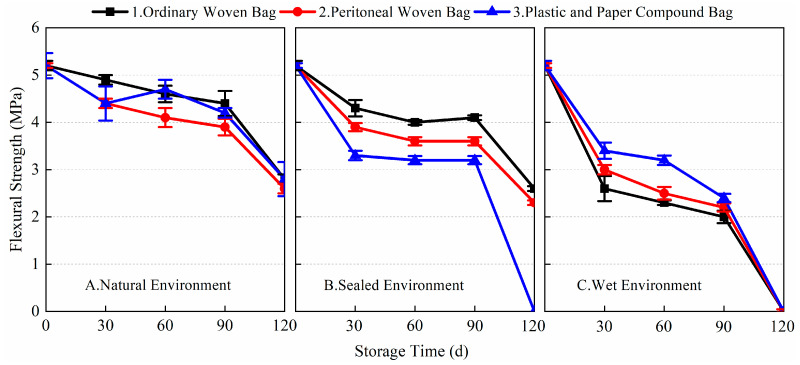
The 1-day flexural strength of finished BMSC with different packaging methods and storage conditions.

**Figure 10:**
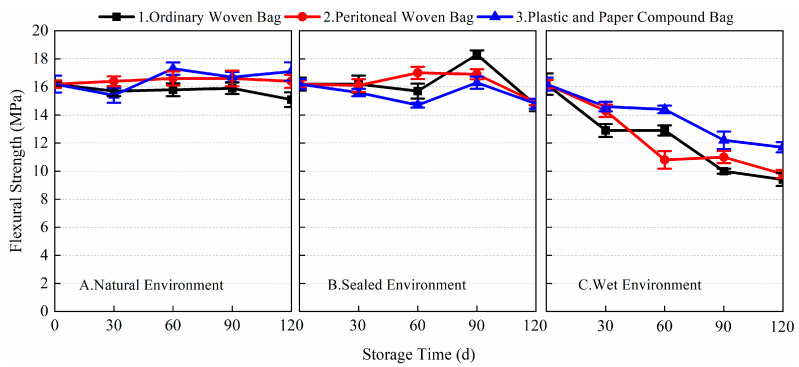
The 28-day flexural strength of finished BMSC with different packaging methods and storage conditions.

**Figure 11:**
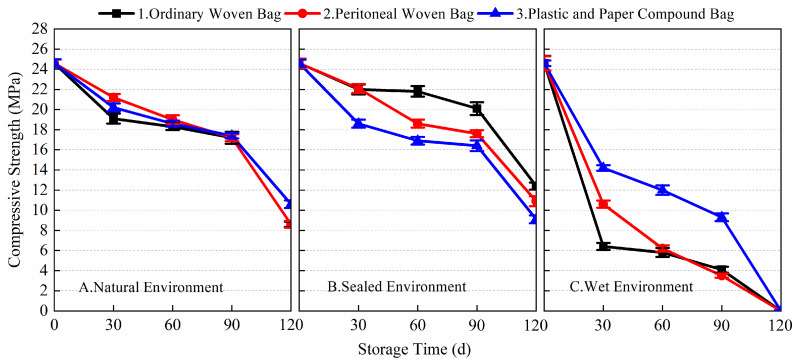
The 1-day compressive strength of finished BMSC with different packaging methods and storage conditions.

**Figure 12:**
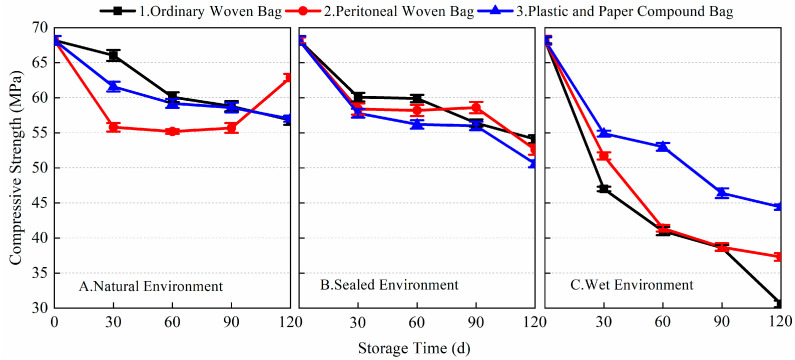
The 28-day compressive strength of finished BMSC with different packaging methods and storage conditions.

Figure 9, Figure 10, Figure 11 and Figure 12 show the data for the 1-day and 28-day mechanical strength of the finished BMSC mortar specimens with storage age using different packaging methods and storage environments. The 1-day flexural and compressive strengths of the blank samples (raw material stored for 0 days) were 5.2 and 24.6 MPa, respectively; the 28-day flexural and compressive strengths were 16.2 and 68.2 MPa, respectively.

Figure 9 shows the data of the 1-day flexural strength of the finished BMSC with different storage conditions and packaging methods. From the data, it can be seen that the packaging methods and storage conditions had a significant effect on the early flexural strength of the finished BMSC. In a natural environment, within 0~90 days, the 1-day flexural strength of the finished BMSC mortar specimens stored using the three packaging methods gradually decreased, consistent with the pattern of change in the a-MgO content; in a sealed environment, the 1-day flexural strength of the finished BMSC mortar specimens decreased significantly in 30 days, and remained basically unchanged in 30~90 days; and in a wet environment, the 1-day flexural strength of the finished BMSC mortar specimens decreased significantly after 30 days of storage, and the decrease in the 1-day flexural strength of the finished BMSC mortar specimens decreased with the prolongation of storage time, but the decrease in the 1-day flexural strength increased again after 90 days. The 1-day flexural strengths of the finished BMSC-A1, A2, A3, B1, B2, B3, C1, C2, and C3 were 4.4, 3.9, 4.2, 4.1, 3.6, 3.2, 2.0, 2.2, and 2.4 MPa, respectively, after 90 days of storage. After 120 days, the 1-day flexural strength of the finished BMSCs with different packaging methods decreased significantly under all three environmental conditions, and the value of the flexural strength could no longer be measured in a wet environment. In natural and sealed environments, the early flexural strength of the finished BMSC stored in ordinary woven bags was better than that of the finished BMSC stored in peritoneal woven bags and plastic and paper compound bags, while, in a wet environment, the early flexural strength of the finished BMSC stored in plastic and paper compound bags was better than that of the finished BMSC stored in peritoneal woven bags and ordinary woven bags.

Figure 10 shows the data for the variation in the 28-day flexural strength of the finished BMSC with storage age for different storage conditions and packaging methods. As shown in the figure, compared with the blank samples, the 28-day flexural strength of the finished BMSC in natural and sealed environments remained almost unchanged with the increase in storage age. The flexural strength even increased slightly after 90 days of storage in the sealed environment, while, in the wet environment, the 28-day flexural strength of the finished BMSC gradually decreased with the extension of storage time. When stored for 90 days, the 28-day flexural strength of the finished BMSC-A1, A2, A3, B1, B2, B3, C1, C2, and C3 were 15.9, 16.6, 16.7, 18.3, 16.9, 16.3, 10.0, 11.0, and 12.2 MPa, respectively. After 120 days of storage, the flexural strengths corresponding to the three packaging methods under wet conditions decreased by 42.0, 39.5, and 27.8%, respectively, compared with the blank samples. The experimental results showed that the storage conditions, packaging methods, and storage age had a more significant effect on the early flexural strength of the finished BMSC, while there was almost no effect on the 28-day flexural strength of the finished BMSC under the conditions of natural environment and sealed environment.

Figure 11 and Figure 12 show the changes in the 1-day and 28-day compressive strength of the finished BMSC mortar specimens with storage age using different environments and packaging methods. From the data, it can be seen that the storage environment, packaging method, and storage age all had a greater impact on the compressive strength of the finished BMSC. As shown in Figure 11, the early compressive strength of the finished BMSC gradually decreased with the extension of storage time. Under the conditions of a natural environment and sealed environment, the early compressive strength of the finished BMSC decreased more slowly in 90 days, while it decreased more significantly in 90~120 days. When stored for 90 days, the decrease in BMSC 1-day compressive strength using the three packaging methods in a natural environment was 30.1, 30.1, and 29.3%, respectively; the decrease in BMSC 1-day compressive strength using the three packaging methods in a sealed environment was 18.3, 28.5, and 33.3%, respectively; and in a wet environment, the 1-day compressive strength of the finished BMSC decreased significantly in 30 days. With the extension of storage time, the compressive strength decreased, and the decrease in the 1-day compressive strength of the finished BMSC using the three kinds of packaging methods when stored for 90 days was 83.3, 85.8, and 62.2%. The 1-day compressive strengths of the finished BMSC-A1, A2, A3, B1, B2, B3, C1, C2, and C3 after 30 days of storage were 19.1, 21.2, 20.2, 22, 22.1, 18.6, 6.4, 10.6, and 14.2 MPa, respectively.

Figure 12 shows the 28-day compressive strength data for the finished BMSC mortar specimens in different environments using the three packaging methods. From the data, it can be seen that in the natural environment and sealed environment, with the extension of storage age, the finished BMSC 28-day compressive strength decline was more moderate, while, in the wet environment, the finished BMSC 28-day compressive strength decline was significant. After 30 days of storage, the 28-day compressive strengths of the finished BMSC-A1, A2, A3, B1, B2, B3, C1, C2, and C3 were 66.04, 55.8, 61.6, 60.1, 58.4, 57.8, 47, 51.7, and 54.9 MPa, respectively. When stored in a natural environment for 90 days, the decreases in the finished BMSC 28-day compressive strength using the three packaging methods were 13.8, 18.3, and 14.1%, respectively. When stored in a sealed environment for 90 days, the decreases in the finished BMSC 28-day compressive strength using the three packaging methods were 17.3, 14.1, and 17.9%, respectively; when stored in a wet environment for 90 days, the decreases in the finished BMSC 28-day compressive strength were 43.4, 43.3, and 32.0%, respectively. The results showed that the storage conditions and packaging methods had a more significant effect on the early compressive strength of the finished BMSC; the effect on the late compressive strength was relatively small, and the appropriate storage time was 90 days. Combined with the results for flexural strength, it can be seen that it was appropriate to use ordinary woven bags to store finished BMSC in a natural environment and sealed environment, the mechanical properties of which were better than those of the peritoneal woven bags and plastic and paper compound bags. In a wet environment, plastic and paper compound bags were required for encapsulation.

### 3.5. Mechanism of the Effect of Packaging Method and Storage Environment on the Activity of MgO

**Figure 13:**
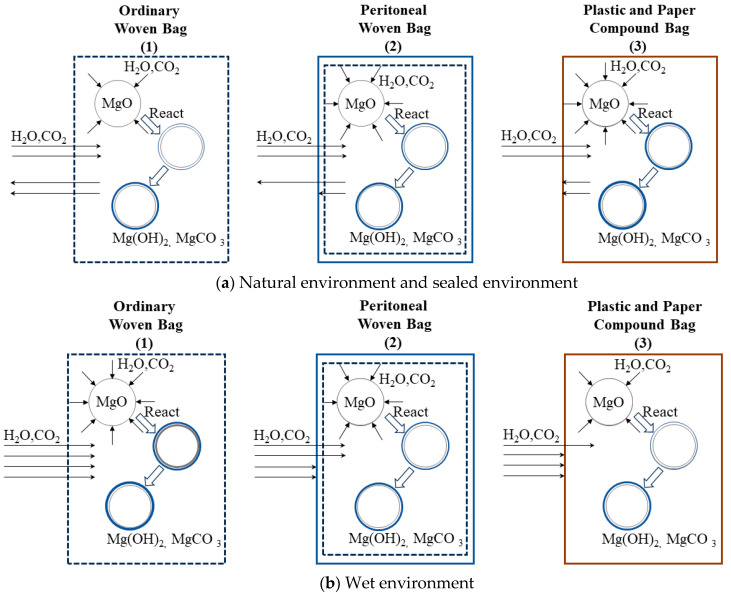
Mechanism of action of storage environment and packaging method on the effect of MgO activity.

Figure 13 shows the analysis of the mechanism of MgO activity in different packaging and storage conditions. Combined with the above research data and the schematic diagram in Figure 13, in the natural environment and sealed environment, the ordinary woven bag was favorable to the exchange of moisture and carbon dioxide inside and outside the bag compared with the peritoneal woven bag and plastic and paper compound bag, which reduced the possibility of moisture absorption and carbonization of the MgO. The peritoneal woven bags and plastic and paper compound bags had good sealing, which was not conducive to internal moisture and carbon dioxide discharge, where light-burned MgO is more prone to moisture absorption and carbonization. Therefore, in the natural environment, BMSC packaged in ordinary woven bags was more conducive to ensuring the mechanical properties of the BMSC. In the wet environment, moisture and carbon dioxide contents were high, and ordinary bag pores allowed a lot of moisture and carbon dioxide inside, causing MgO hydrolysis or carbonization. Plastic and paper compound bags and peritoneal woven bags had better airtightness than ordinary woven bags and could better prevent moisture and carbon dioxide from entering, thereby reducing the potential for light-burned MgO reactions with water and carbon dioxide. Therefore, in a wet environment, plastic and paper compound bag and peritoneal woven bag encapsulation are more advantageous for storing light-burned MgO and BMSC.

## 4. Conclusions

In the sealed environment, the loss of a-MgO content in the light-burned MgO was smaller, which was favorable to the hydration reaction of BMSC. In the wet environment, the loss of a-MgO content was greater. In the natural environment, the change in the a-MgO content of the light-burned MgO was between the two. The setting time of BMSC was closely related to the a-MgO content in the light-burned MgO. The change in the a-MgO content was smaller in the sealed environment, and the increase in the BMSC setting time was smaller. This is favorable to ensuring construction efficiency.

In the natural and sealed environments, the packaging method and storage age had a more significant effect on the early mechanical strength of BMSC but a smaller effect on its 28-day mechanical strength. In the natural environment, the light-burned MgO or BMSC stored in ordinary woven bags could be stored for 90 days; in the wet environment, the light-burned MgO or BMSC stored in plastic and paper compound bags could be stored for less than 30 days.

In natural and sealed environments, BMSC can be packaged in ordinary woven bags, which is more conducive to water exchange and reduces the possibility of moisture absorption and carbonization. This is more economical for engineering applications. The performance of plastic and paper compound bags in wet environments is more excellent compared with that of ordinary woven bags and peritoneal woven bags, which can better prevent water and carbon dioxide from entering the bag, ensuring its mechanical properties.

## Figures and Tables

**Table 1:** Chemical composition of raw materials/%.

Raw Material	Mg^2+^	SO_4_^2−^	Ca^2+^	SiO_2_	Al_2_O_3_	Fe_2_O_3_	CaO	MgO	SO_3_	Na_2_O	K_2_O	Loss on Ignition
MgSO_4_·7H_2_O	9.82	39.92	0.02									
MgO				0.30	0.26	0.51	2.15	79.67	0.23	0.23	0.24	16.41
FA				54.18	28.78	7.88	1.92	0.66	0.35	0.54	0.78	0.32

**Table 2:** Sample labeling.

	Packaging Method	Ordinary Woven Bag	Peritoneal Woven Bag	Plastic and Paper Compound Bag
Storage Conditions				
Natural Environment		A1	A2	A3
Sealed Environment		B1	B2	B3
Wet Environment		C1	C2	C3

## Data Availability

All data generated or analyzed during this study are included in this article. All data included in this study are available upon request by contact with the corresponding author.
